# Diagnostic value of mucosal-associated invariant T+ cells combined with inflammatory factors in differentiating chronic rhinosinusitis subtypes with or without nasal polyps

**DOI:** 10.5937/jomb0-59840

**Published:** 2026-01-28

**Authors:** Yilong Wang, Yongjun Feng, Jie Song, Zhonglin Mou

**Affiliations:** 1 The Second Affiliated Hospital of Hainan Medical University, Department of Otolaryngology, Head & Neck Surgery, Haikou, China

**Keywords:** mucosal-associated invariant T cells, chronic rhinosinusitis, nasal polyps, inflammatory factors, biomarker, mukozno-asocirane invarijantne T celije, hronični rinosinusitis, nazalni polipi, inflamatorni faktori, biomarker

## Abstract

**Background:**

The present study attempted to analyze the effect of mucosal-associated invariant T (MAIT) combined with inflammatory factors in identifying chronic rhinosinusitis (CRS) without nasal polyps (CRSsNP) and CRS with nasal polyps (CRSwNP) that provides a clinical reference.

**Methods:**

In this retrospective study, we enrolled 56 CRSsNP patients, 68 CRSwNP patients, and 64 healthy controls. Flow cytometry was used to detect the frequency of peripheral blood MAIT+ cells (MAIT+). Serum levels of hs-CRP, IL-ip, and IL-6 were measured by ELISA and automatic blood cell analyzer, and their correlations with MAIT cell alterations were analyzed. The diagnostic effect of MAIT+ combined with hs-CRP IL-ip, and IL-6 on CRS was analyzed using ROC curves.

**Results:**

Both CRSsNP and CRSwNP patients exhibited significantly reduced MAIT+ frequencies compared to controls (P&lt; 0.05), with CRSwNP showing the most pronounced decrease (P &lt; 0.0 5). MAIT+ frequency was inversely correlated with hs-CRP IL-ip, and IL-6 in CRS patients. ROC analysis demonstrated that MAIT+ effectively discriminated CRSsNP (AU C= 0.793) and CRSwNP (AU C= 0.863) from healthy individuals (P&lt; 0.01). After combined with inflammatory factors, the diagnostic effect of MAIT+ combined inflammatory factors on CRSsNP (AU C= 0.933) and CRSwNP (AU C= 0.939) was further improved.

**Conclusions:**

Detection of MAIT+ combined with inflammatory factors may provide potential biomarkers for CRS subtype identification.

## Abstract (sr)

**Uvod:**

U ovoj studiji pokušano je da se analizira efekat mukozno-asociranog invarijantnog T (MAIT) u kombinaciji sa inflamatornim faktorima u identifikaciji hroničnog rinosinusitisa (CRS) bez nosnih polipa (CRSsNP) i CRS sa nosnim polipima (CRSwNP), što pruža kliničku referencu.

**Metode:**

U ovoj retrospektivnoj studiji, uključili smo 56 pacijenata sa CRSsNP, 68 pacijenata sa CRSwNP i 64 zdrave kontrolne osobe. Protočna citometrija je korišćena za detekciju učestalosti MAIT+ ćelija u perifernoj krvi (MAIT+). Serumski nivoi hs-CRP IL-ip i IL-6 mereni su ELISA testom i automatskim analizatorom krvnih zrnaca, a analizirane su njihove korelacije sa promenama MAIT ćelija. Dijagnostički efekat MAIT+ u kombinaciji sa hs-CRP IL-ip i IL-6 na CRS analiziran je korišćenjem ROC krivih.

**Rezultati:**

I pacijenti sa CRSsNP i CRSwNP pokazali su značajno smanjenu učestalost MAIT+ u poređenju sa kontrolnom grupom (P&lt; 0,05), pri čemu je CRSwNP pokazao najizraženije smanjenje (P&lt; 0,05). Učestalost MAIT+ je bila obrnuto korelirana sa hs-CRP, IL-ip i IL-6 kod pacijenata sa CRS. ROC analiza je pokazala da MAIT+ efikasno razlikuje CRSsNP (AU C= 0,793) i CRSwNP (AU C= 0,863) od zdravih osoba (P &lt; 0 ,0 i). Nakon kombinovanja sa inflamatornim faktorima, dijagnostički efekat MAIT+ kombinovanih inflamatornih faktora na CRSsNP (AU C= 0,933) i CRSwNP (AU C= 0,939) je dodatno poboljšan.

**Zaključak:**

Detekcija MAIT+ u kombinaciji sa inflamatornim faktorima može pružiti potencijalne biomarkere za identifikaciju podtipa CRS.

## Introduction

Chronic rhinosinusitis (CRS) represents a prevalent chronic inflammatory disorder in otorhinolaryngology, affecting approximately 8.71% of the global population [1]. CRS without nasal polyps (CRSsNP) and CRS with nasal polyps (CRSwNP) are the two most common subtypes of CRS, and although their clinical manifestations are basically the same, there are significant differences in pathogenesis, immunologic features, and therapeutic responses [2]. Patients with CRSwNP are often associated with Th2-type inflammation-dominated polyp formation and better response to biologic agents, whereas CRSsNP is more characterized by neutrophilic infiltration and a mixed Th1/Th17 inflammatory phenotype, and is less responsive to conventional therapy [3]. This heterogeneity suggests that distinct immune regulatory networks may exist in different subtypes, and resolving the differences in their key immune cells can help to reveal the nature of the disease and guide precision therapy.

In recent years, mucosal-associated invariant T (MAIT)^+^ cells (MAIT^+^), as »bridge cells« between innate and adaptive immunity, have attracted significant attention for their role in mucosal immune regulation [4]. These unconventional T lymphocytes predominantly reside in mucosal barrier tissues such as the nasal epithelium, where they detect microbial metabolites through the MHC class I-related gene 1 (MR1)-dependent pathway and rapidly orchestrate immune responses via the production of effector cytokines like Interleukin (IL)-17 and Interferon-γ (IFN-γ) [5]. Intriguingly, the core pathological features of CRS-mucosal barrier disruption and persistent microbial dysbiosis - are closely linked to the established biological characteristics of MAIT^+^ [6]. This compelling association positions MAIT^+^ as potential key players in CRS pathogenesis. Notably, MAIT^+^ demonstrate functional plasticity with dichotomous immunoregulatory capacity: while their production of IL-17 may drive tissue inflammation, concomitant IL-10 secretion can mediate immunosuppression [7]. Chiba et al. documented that MAIT cell depletion in systemic lupus erythematosus correlates with elevated proinflammatory cytokines [IL-6 and Tumor necrosis factor-α (TNF-α)] [8], suggesting that MAIT^+^ may influence disease outcomes by modulating the balance of inflammatory cytokines. More recently, Jiang Y et al. [9], highlighted the association of MAIT^+^ cells cells with allergen immunotherapy in allergic rhinitis, suggesting the important clinical significance of MAIT^+^ in CRS. Nevertheless, the dynamic changes of peripheral blood MAIT^+^ in CRS patients and their interaction with key inflammatory cytokines remain unexplored, leaving a critical knowledge gap impeding our understanding of immune dysregulation in CRS.

The aim of this study is to systematically compare the differences in peripheral blood MAIT between CRSwNP and CRSsNP patients, and analyze the diagnostic effect of MAIT^+^ combined with inflammatory factors on CRS. The results of this study will not only enrich the understanding of the immunopathological mechanisms of CRS, but also lay a theoretical foundation for the development of novel diagnostic and therapeutic protocols based on the regulation of MAIT^+^.

## Materials and methods

### Study population

We conducted a retrospective analysis of CRS patients admitted to our hospital between January 2024 and January 2025. Based on sample size estimation using G*Power [tail(s) = 2, α = 0.05, effect size=0.5, power=0.95, 10% risk of dropout], we enrolled 124 participants, including 56 cases of CRS without nasal polyps (CRSsNP) and 68 cases of CRS with nasal polyps (CRSwNP). Additionally, 60 age- and sex-matched healthy individuals undergoing routine health examinations during the same period were included as controls. The study protocol was approved by the Ethics Committee of our hospital. As this was a retrospective study, the requirement for informed consent was waived.

Inclusion Criteria: (1) Confirmed diagnosis of CRS according to established criteria [2]; (2) Age 18 years; (3) Complete clinical records available; (4) Disease duration 1 year with stable chronic disease status; (5) No history of endoscopic sinus surgery or treatment with corticosteroids/immunosuppressants within the preceding 3 months.

Exclusion Criteria: (1) Comorbid immune disorders such as asthma or allergic rhinitis; (2) Active or historical malignancy; (3) Evidence of active infections at enrollment; (4) Metabolic syndrome or hepatic/renal dysfunction; (5) Smoking history 10 pack-years; (6) Alcohol consumption >40 g/day; (7) Pregnant or lactating women.

### MAIT cell detection

Peripheral venous blood (10 mL) was collected from fasting subjects using heparin anticoagulation. The blood was diluted with an equal volume of PBS, layered over Ficoll-Paque separation medium, and centrifuged (2000 × g, 30 min) to isolate peripheral blood mononuclear cells (PBMCs). PBMCs (1 × 10^6^/tube) were then stained with fluorochrome-conjugated antibodies against MAIT-APC [MAIT^+^ were stained with FITC-anti-Vα7.2 (clone 3C10, BioLegend) and PE-anti-CD161 (clone HP-3G10, BioLegend)]. Antibody incubation was performed in the dark at 4°C for 30 minutes. Cells were acquired on a BD FACSCanto II flow cytometer (BD Biosciences) with voltages optimized using unstained and fluorescence-minus-one controls. Compensation was performed using single-stained beads.

### Inflammatory cytokine measurement

Serum was obtained by allowing peripheral blood to clot at room temperature for 30 min, followed by centrifugation (1500 × g, 10 min). Levels of IL-1β and IL-6 were measured using Enzyme-Linked Immunosorbent Assay (ELISA) kits (Shanghai Jianglai Biotechnology Co., Ltd.) according to the manufacturer's protocols. Standard substance: the standard curve substance of the kit (containing known concentrations of hs-CRP/IL-1β/IL-6) was used to draw the standard curve. Quality control materials: internal quality control materials (high, medium and low concentration, matched with sample matrix) and external quality assessment (EQA) samples. Serum samples were added to 96-well plates at gradient dilutions (0, 10, 50, 100, 200, 500 pg/mL) according to the manufacturer's instructions, with 100 μL per well. After incubation at 37°C for 90 min, 100 μL of enzyme-labeled secondary antibody was added to each well after washing and incubated at 37°C for 90 min. After washing, 100 μL TMB substrate solution was added to each well, and incubated at 37°C in the dark for 15-30 min. Then 50 μL termination solution was added to each well, and the absorbance (OD) value of each well was detected by a microplate reader (Tecan Spark) at 450 nm wavelength. ELISA kits had a sensitivity of <1 pg/mL for IL-1β/IL-6 and <0.1 mg/L for hs-CRP Intra-assay CV was <8% and inter-assay CV <10% (manufacturer's specifications).

Hypersensitive-C reactive protein (hs-CRP) was detected by automatic blood cell analyzer (Mindray BC-5800). The results were detected automatically after the samples were loaded on the machine. The reaction reagent was latex particle suspension containing anti-human hs-CRP monoclonal antibody. The diluent was a low ionic strength buffer. Calibrators were matched calibrators (containing known concentrations of hs-CRP ranging from the lower limit of detection to the upper limit of linearity).

### Outcome measures

Primary indicator: Differences in MAIT cell frequency among the three groups of study subjects, specifically the proportion of MAIT^+^, and the discriminatory efficacy of MAIT^+^ for CRS. Secondary indicators: The relationship between MAIT^+^ and inflammatory cytokine levels.

### Statistical methods

All analyses were conducted using SPSS version 26. Continuous variables with a normal distribution (verified by the Shapiro-Wilk test) are presented as (χ̄±s) and analyzed by one-way analysis of variance with Bonferroni post-hoc adjustment. Categorical data are reported as counts (percentages) and compared using chi-square tests. Post-hoc comparisons used Bonferroni correction. Adjusted P-values are reported unless otherwise specified. Diagnostic performance was evaluated through receiver operating characteristic (ROC) curve analysis. Statistical significance was set at P<0.05.

## Results

### Comparison of clinical data

The three groups demonstrated comparable baseline clinical characteristics, with no significant intergroup differences in age, sex distribution, or other baseline parameters (P>0.05). Importantly, disease duration showed no statistical variation between CRSsNP and CRSwNP groups (P>0.05), ensuring balanced group characteristics for subsequent analyses. However, the Lund-Mackay scores were higher in the CRSwNP group than in the CRSsNP group (P<0.001), which was due to the occurrence of nasal polyps that led to sinus obstruction, and therefore the Lund-Mackay scores of the CRSwNP patients were naturally higher (Table 1).

**Table 1:** Baseline characteristics of the study population. Note:* indicates P<0.05 compared with the control group, # indicates P<0.05 compared with the CRSsNP group. Lund-Mackay score (10): Scores ranged from 0 to 24, with higher scores indicating more severe lesions.

Groups	Control (n=64)	CRSsNP (n=56)	CRSwNP (n=68)	Statistics	P
Age	45.65±7.24	46.80±6.97	45.74±6.27	F=0.519	0.596
Sex				2=1.333	0.514
Male	38 (59.38%)	29 (51.79%)	42 (61.76%)		
Female	26 (40.63%)	27 (48.21%)	26 (38.24%)		
Family History of CRS				2=0.435	0.805
Yes	6 (9.38%)	6 (10.71%)	5 (7.35%)		
No	58 (90.63%)	50 (89.29%)	63 (92.65%)		
Ethnicity				2=0.509	0.775
Han nationality	60 (93.75%)	51 (91.07%)	64 (94.12%)		
Minority nationality	4 (6.25%)	5 (8.93%)	4 (5.88%)		
Smoking				2=0.305	0.859
Yes	26 (40.63%)	20 (35.71%)	26 (38.24%)		
No	38 (59.38%)	36 (64.29%)	42 (61.76%)		
Drinking				2=0.893	0.640
Yes	20 (31.25%)	14 (25.00%)	22 (32.35%)		
No	44 (68.75%)	42 (75.00%)	46 (67.65%)		
BMI (kg/m^2^)	22.47±2.55	22.71±1.50	23.04±1.74	F=1.377	0.255
SBP (mmHg)	105.73±7.97	10 5.8 4±9.1 5	103.40±7.43	F=1.838	0.162
DBP (mmHg)	76.30±6.53	76.20±6.67	74.26±6.93	F=1.874	0.157
Comorbidities					
Diabetes	14 (21.88%)	14 (25.00%)	16 (23.53%)	2=0.164	0.921
Hypertension	10 (15.63%)	8 (14.29%)	12 (17.65%)	2=0.267	0.875
Hyperlipidemia	9 (14.06%)	11 (19.64%)	11 (16.18%)	2=0.683	0.711
Occupational exposures (dust,<br>chemicals, allergens)				2=1.032	0.597
Yes	19 (29.69%)	21 (37.50%)	25 (36.76%)		
No	45 (70.31%)	35 (62.50%)	43 (63.24%)		
Duration of CRS (weeks)	-	26.89±6.95	25.91±7.76	t=0.734	0.464
Lund-Mackay score	-	10.11±1.87	16.04±4.30	t=9.596	<0.001

### Comparison of serum inflammatory factors

In terms of inflammatory factors, we found that hs-CRP IL-1β, and IL-6 were significantly higher in CRS patients than in the control group (P<0.05). However, there was no difference in hs-CRP IL-1β, and IL-6 between the CRSwNP and CRSsNP groups (P>0.05) (Table 2).

**Table 2:** Comparison of serum inflammatory factors. Note:* indicates P<0.05 compared with the control group.

Groups	Control (n = 64)	CRSsNP (n = 56)	CRSwNP (n = 68)	F	P
hs-CRP (mg/L)	5.72±1.96	15.55±3.65*	14.86±3.21*	199.204	<0.001
IL-1β (pg/mL)	17.65±6.78	28.96±5.27*	29.02±7.11*	61.812	<0.001
IL-6 (pg/mL)	12.86±4.77	24.40±5.01*	24.54±6.28*	92.073	<0.001

### Comparison of MAIT^+^ and relation to serum inflammatory factors

**Figure 1:**
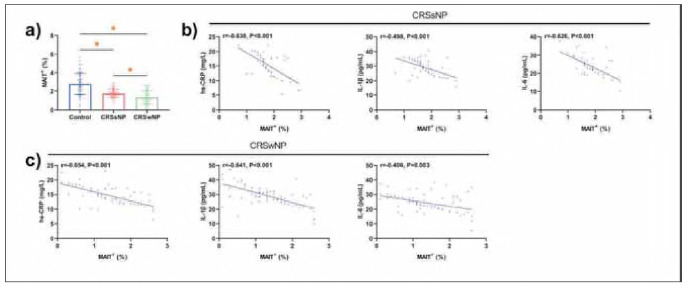
Comparison of MAIT^+^ and correlation with serum inflammatory factors. a) Comparison of MAIT^+^, b) Correlation analysis between MAIT^+^ and serum inflammatory factors in patients in the CRSsNP group, c) Correlation analysis between MAIT^+^ and serum inflammatory factors in patients in the CRSwNP group. *P < 0.05.

We compared MAIT cell frequencies among the three groups. The control group exhibited a baseline MAIT^+^ frequency of (2.79±1.15)%, reflecting a normal steady state. In contrast, both the CRSsNP and CRSwNP groups showed reduced MAIT^+^ frequencies - (1.76±0.44)% and (1.26±0.60)%, respectively - with the CRSwNP group demonstrating an even lower proportion (P<0.05). Pearson correlation analysis showed MAIT^+^ frequencies exhibited inverse correlations with inflammatory factors, suggesting a potential bidirectional interaction requiring mechanistic validation (P<0.001) (Figure 1).

### Analysis of the role of MAIT^+^ in the assessment of CRS

**Figure 2:**
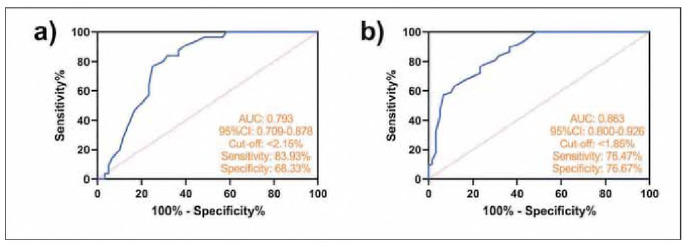
Analysis of the role of MAIT^+^ in the assessment of CRS. a) ROC curve of MAIT^+^ for diagnosis of CRSsNP occurrence, b) ROC curve of MAIT^+^ for diagnosis of CRSwNP occurrence.

ROC curve analysis using the MAIT^+^ test results of the control group versus the CRSsNP group showed that when MAIT^+^<2.15%, the sensitivity for detecting CRSsNP patients was 83.93% and the specificity for distinguishing them from healthy controls was 68.33% (P<0.001, AUC=0.793). In contrast, the analysis of the MAIT^+^ test results using the control and CRSwNP groups showed that the sensitivity and specificity of MAIT^+^ to diagnose CRSwNP were 76.47% and 76.67%, respectively (P<0.001, AUC=0.863) for MAIT^+^<1.85% (Figure 2).

### The diagnostic effect of MAIT^+^ combined inflammatory factors on CRSwNP or CRSsNP

**Figure 3:**
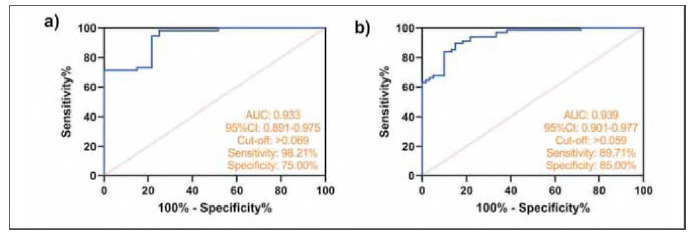
The diagnostic effect of MAIT^+^ combined inflammatory factors on CRSwNP or CRSsNPa) ROC curve of MAIT^+^ combined with inflammatory factors in the diagnosis of CRSsNP b) ROC curve of MAIT^+^ combined with inflammatory factors in the diagnosis of CRSwNP.

Finally, we established a combined diagnostic model for CRSwNP and CRSsNP according to the results of MAIT^+^ and inflammatory factors (the combined model was the regression coefficient establishment of MAIT, hs-CRP IL-1β, and IL-6 affecting CRSwNP or CRSsNP). ROC curve analysis showed that when MAIT^+^ inflammatory factors were used, the AUC for diagnosis of CRSwNP increased to 0.933 and the AUC for diagnosis of CRSnNP increased to 0.939 (P<0.001, Figure 3). Compared with MAIT^+^ alone, it has a significant improvement and a higher clinical reference value.

## Discussion

This study represents the first comprehensive investigation into the heterogeneous characteristics of MAIT^+^ in peripheral blood across distinct CRS subtypes (CRSsNP and CRSwNP) while elucidating their dynamic interplay with inflammatory cytokine networks. These findings provide novel mechanistic insights into immune dysregulation in CRS pathogenesis [10].

hs-CRP IL-1β, and IL-6 are inflammatory factors that are often used in clinical practice to evaluate the degree of inflammatory response in CRS [11] [12]. In the present study, it was seen that hs-CRP IL-1β, and IL-6 were elevated in the peripheral blood of patients with CRS compared with the control group, which again validated the relationship between these inflammatory factors and CRS. However, hs-CRP IL-1β, and IL-6 levels did not show significant differences between the CRSwNP and CRSsNP groups, which shows that these traditional inflammatory factors in the peripheral blood, although capable of assessing the progression of CRS, were not able to discriminate between CRSwNP and CRSsNP. This result also once again emphasizes the search for specific clinical markers for the assessment of CRS disease importance. It is important to note that although there is no difference in the levels of these inflammatory factors in peripheral blood, there may be differences in the expression of local tissues or specific cell sources. However, this is beyond the scope of our study, and we will conduct additional analyses in future studies. Based on this situation, we targeted the detection and analysis of MAIT^+^ in CRS. We observed a significant reduction in peripheral blood MAIT^+^ proportions in both CRSsNP and CRSwNP patients compared to healthy controls, suggesting a potentially significant link between MAIT^+^ and the progression of CRS. This phenomenon may be driven by multiple mechanisms: (1) Tissue migration: MAIT^+^ express chemokine receptors such as CCR6 and CXCR6, enabling them to respond to local inflammatory signals (e.g., CCL20) in the nasal mucosa and migrate to lesion sites to participate in immune responses [13] [14]. This hypothesis is supported by studies showing increased MAIT cell infiltration in nasal polyp tissues [15]. (2) Chronic activation-induced exhaustion: Chronic stimulation by microbial and damage-associated molecular patterns in the CRS microenvironment may drive the overexpression of inhibitory receptors such as PD-1 and TIM-3, leading to MAIT cell functional exhaustion [16]. (3) Th2 microenvironment suppressing MAIT cell generation: The overexpression of inflammatory cytokines in CRS patients may inhibit the differentiation of bone marrow hematopoietic stem cells or thymic output, thereby reducing the peripheral homeostasis of MAIT^+^ [17]. In CRS patients, lower frequency of MAIT^+^ were seen in the CRSwNP group, suggesting that the pathologic significance of MAIT^+^ is more prominent in the concomitant polyp subtype, which may be related to local recruitment or accelerated apoptosis of MAIT^+^ in the polyp microenvironment. Notably, we found a negative correlation between MAIT^+^ and serum inflammatory factors in the CRSwNP and CRSsNP groups, confirming a potential link between MAIT^+^ and serum inflammatory factors and suggesting that MAIT^+^ may be involved in the progression of CRS by regulating the release of inflammatory factors, and that their dysfunction may exacerbate local or systemic inflammatory responses.

Further analysis of the ROC curve showed that MAIT^+^ frequency could not only distinguish CRS patients from healthy people (AUC=0.793-0.863), but also further improve the diagnostic effect for CRSsNP or CRSwNP when combined with inflammatory factors (AUC=0.933-0.939). This finding provides a reliable reference value for clinical differentiation of CRSsNP and CRSwNP in the future. We believe that the advantages of MAIT^+^ combined inflammation in the diagnosis of CRSsNP and CRSwNP are mainly reflected in the following: (1) Subtype specificity. Conventional inflammatory factors reflect systemic or local inflammatory intensity but are unable to differentiate CRS subtypes [18], whereas the stepwise decrease in MAIT^+^ may be related to its specific regulatory role in Th1/Th2/Th17 immune imbalance [19]. (2) Dynamic sensitivity, the degree of MAIT^+^ depletion correlates with the process of disease chronicity or polyp formation, and may serve as a potential marker for disease staging. (3) Independent of acute inflammatory disturbances. hs-CRP is susceptible to infections or acute exacerbations, whereas a decrease in MAIT^+^ frequency is more likely to reflect the state of long-term immune disorders [20], which makes it suitable for the assessment of the stabilization phase of chronic diseases.

However, the present study was a single-center cross-sectional study with a small sample size and did not include patients with different disease stages. Therefore, it was not possible to assess the dynamic relationship between changes in MAIT cell frequency and the evolution of disease activity or severity. The specific mechanisms underlying the reduced MAIT^+^ frequency (e.g., migration, apoptosis, or epigenetic modulation) have not been validated by in vitro experiments or animal models. Finally, the association between peripheral blood and local immunity could not be clarified because MAIT cell infiltration in nasal mucosa or polyp tissues was not simultaneously detected. Flow cytometric MAIT^+^ cell detection requires specialized equipment and expertise, limiting accessibility in resource-limited settings. Subsequently, we need to conduct additional studies to address the above limitations.

## Conclusion

The frequency of peripheral blood MAIT^+^ represents a novel biomarker. When combined with serum inflammatory factors (hs-CRP IL-1β, IL-6), it can accurately distinguish between CRS subtypes (CRSsNP and CRSwNP). In the future, it is necessary to promote the standardization and clinical translation of MAIT^+^ through multidisciplinary collaboration, and at the same time to explore its central role in CRS immunoregulation, so as to provide a theoretical basis for the development of precise diagnostic and treatment strategies.

## Dodatak

### Availability of data and materials

The data that support the findings of this study are available from the corresponding author upon reasonable request.

The data used to support the findings of this study are available from the corresponding author upon request.

### Funding

This study was supported by the Hainan Provincial Natural Science Foundation Youth Fund Project (No.824QN389).

### Acknowledgements

Not applicable.

### Conflict of interest statement

All the authors declare that they have no conflict of interest in this work.
